# Brugada Sign in a Cardiac Transplant Donor

**DOI:** 10.7759/cureus.27619

**Published:** 2022-08-02

**Authors:** Issa Asfour, Akil A Sherif, Pradnya Brijmohan Bhattad, Ajay K Mishra, Nitish Sharma, Mark Kranis

**Affiliations:** 1 Internal Medicine, East Tennessee State University, Johnson City, USA; 2 Cardiovascular Medicine, Saint Vincent Hospital, UMass Chan Medical School, Worcester, USA; 3 Cardiovascular Medicine, Saint Vincent Hospital, Worcester, USA

**Keywords:** sudden cardiac death, cocaine, heart donor, cardiac transplant, brugada syndrome, brugada sign

## Abstract

Brugada syndrome (BrS) is a rare entity represented by the Brugada sign on an electrocardiogram (EKG) and is associated with sudden cardiac death (SCD). There is little data to guide the management of donor Brugada syndrome in the setting of cardiac transplantation.

A 31-year-old male sustained out-of-hospital cardiac arrest secondary to polysubstance use and was found asystole. Bystander cardiopulmonary resuscitation (CPR) with advanced cardiovascular life support (ACLS) protocol was initiated. Return of spontaneous circulation (ROSC) was achieved and the patient was taken to the emergency room (ER) in sinus rhythm with an initial presenting EKG showing the Brugada sign. A toxicological screen for cocaine was positive. The patient was eventually declared brain dead and underwent angiographic and echocardiographic evaluation as a donor heart for cardiac transplantation and was accepted for transplantation.

Cardiac arrest in a young patient with a Brugada sign on EKG is a concern for BrS. Cocaine exerts a sodium channel blockade that can unmask BrS. Genetic testing for sodium voltage-gated channel alpha subunit 5 (​​​​​​SCN5A) gene mutation was negative, however, only 15% to 30% of patients carry the mutation. We proceeded with cardiac transplantation and suggested an implantable cardioverter defibrillator (ICD) for primary prevention in the recipient, should further specialized testing reveal a continued concern for BrS. We suggest the necessity for further data to guide decisions in patients with BrS undergoing cardiac transplantation.

## Introduction

Brugada syndrome (BrS) affects the heart and is defined by a specific pattern of right bundle branch block (RBBB) with coved or sometimes saddle-back ST-segment elevation in leads V1-V3, in addition to an absence of underlying structural heart disease, an increased tendency for ventricular arrhythmia, ventricular fibrillation, and sudden cardiac death [1]. This electrocardiogram (EKG) pattern may also be provoked by certain drug toxicities such as cocaine [2].

There is little data to guide the management of BrS in a heart donor in the context of cardiac transplantation. This report is unique as it describes the case of a patient with BrS who was accepted as a donor for cardiac transplantation. 

This article was previously presented as a meeting abstract at the American College of Cardiology (ACC) Annual Scientific Meeting on April 2, 2022.

## Case presentation

**Figure 1:**
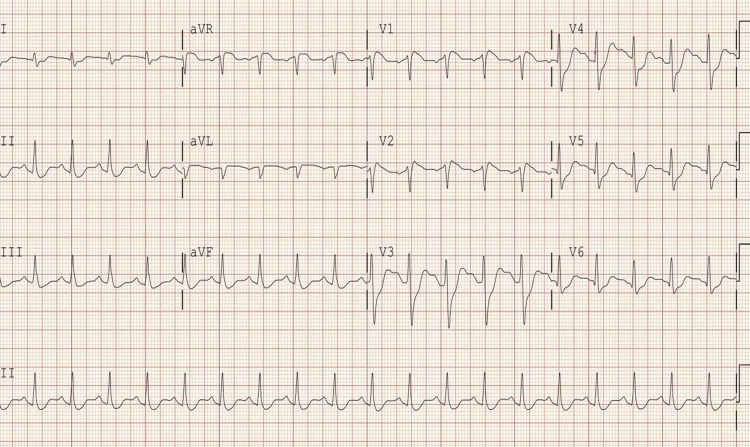
EKG showing Brugada sign-coved ST-segment elevation with T-wave inversion in leads V1-V2 with a positive terminal QRS. EKG: Electrocardiogram

A 31-year-old male sustained out-of-hospital cardiac arrest secondary to polysubstance use and was found asystole. Bystander cardiopulmonary resuscitation (CPR) with advanced cardiovascular life support (ACLS) protocol was initiated and CPR was performed by the bystanders until the arrival of the emergency medical services. Return of spontaneous circulation (ROSC) was achieved after four minutes and the patient was taken to the emergency room (ER) in sinus rhythm with an initial EKG showing a Brugada sign (Figure 1). 

A toxicological screen for cocaine was positive. The patient was closely monitored in the intensive care unit for post-cardiac arrest care and was on mechanical ventilation with no electrolyte disturbances. He did not undergo cardiac catheterization immediately post-cardiac arrest. He received hypothermia protocol per then post-cardiac arrest care guidelines. His neurological function remained poor for several days following which he was eventually declared brain dead by the critical care and neurology teams.

The patient was deemed to be a suitable donor candidate for cardiac transplantation after a thorough discussion with the patient's family and the regional organ bank. Cardiac catheterization for evaluation of the donor's heart was performed and angiographically normal coronary arteries were found. There was no evidence of any structural heart disease based on the transthoracic echocardiogram and coronary angiogram.

The presenting electrocardiogram showed the Brugada sign and therefore further evaluation with genetic testing was pursued. Genetic testing for sodium channel voltage-gated type 5 alpha subunit (SCN5A) gene mutation was negative. The recipient and the regional organ bank and the transplant teams were updated regarding this possibility. After a thorough review, the transplant team proceeded with cardiac transplantation.

## Discussion

Brugada syndrome is most frequently seen in young individuals and it is a genetically determined disease affecting the sodium channel, resulting in reentrant ventricular arrhythmia and sudden cardiac death [3 ]. The Brugada EKG pattern associated with the BrS is subdivided into three types based on the distinct findings in leads V1-V3 (Table 1) [1].

**Table 1:** Types of Brugada patterns on EKG EKG: Electrocardiogram

Type	EKG Findings
Type 1 Brugada pattern	Coved ST-segment elevation ≥2 mm followed by down-sloping concave with a negative symmetric T-wave
Type 2 Brugada pattern	Saddle-back convex ST segment elevation with a variable T-wave in V1
Type 3 Brugada pattern	Similar to type 2 but with a positive or flat T-wave in lead V2

Certain tests can be performed to diagnose BrS, such as genetic testing for SCN5A. However, this genetic test is only positive in 15% to 30% of patients, so it is not mandatory for diagnosis. Other tests can be carried out through the use of sodium channel blocker agents like ajmaline, flecainide, or procainamide to provoke the pattern in patients with inherited cardiac channelopathy [1].

The EKG pattern associated with BrS is transient in 40% of cases, but it can be unmasked by sodium channel blockers such as cocaine [4]. Cocaine intoxication has been previously associated with BrS EKG patterns of types 1 and 2 [5]. The exact mechanism is thought to be due to the unmasking of the underlying genetic myocardial defect, caused by blocking the mutated sodium channels which results in an altered voltage gradient and induces the described EKG pattern [2]. Ventricular arrhythmia resulting in sudden cardiac death in a young individual with no known structural heart disease is always concerning as it might indicate an underlying channelopathy as a differential.

This report describes a unique case of BrS induced by cocaine intoxication in an individual undergoing heart donation. The presence of terminal QRS positivity and coved ST elevation in V1 on the EKG (Brugada sign) in the background of cocaine consumption with a history of the unwitnessed syncopal event and cardiac arrest in a young patient puts BrS at the top of the differential list. Cardiac arrest in the setting of cocaine toxicity is also a differential and these patients rarely manifest the Brugada sign. Arriving at a diagnosis of BrS is challenging but holds significant implications in the setting of cardiac transplantation, as patients with a history of previous ventricular arrhythmia have a 10 % annual risk of recurrence in the first four years, thus, the only proven therapy for BrS is implantable cardioverter-defibrillator (ICD) placement [1].

Current data to guide the management of BrS in the setting of cardiac transplantation are inadequate. The two central concepts in the selection of the donor heart for transplantation are the quality of the donor's heart and the matching of the donor's heart with the recipient's individual needs. The quality of the donor's heart is assessed through standard criteria using serum cardiac enzyme markers, electrocardiogram, echocardiogram, and coronary angiography that do not take into account transient arrhythmias that can be provoked by a certain agent such as cocaine-induced BrS [6].

In our case, an echocardiogram and coronary angiographic evaluation of the donor's heart were performed and found to be normal without any evidence of structural disease, and genetic testing for an SCN5A gene mutation was pursued and found to be negative. While a positive test in this setting may have been suggestive of BrS, a negative test does not rule out the disease given the high degree of genetic heterogeneity associated with BrS. After discussing our findings with the recipient, the transplant teams, and the organ bank, a decision was made to proceed with the cardiac transplantation. It was suggested that the transplant team consider an ICD implantation for primary prevention in the recipient should further tests reveal concerns of BrS.

## Conclusions

Brugada syndrome may be unmasked by drugs causing sodium channel blockade, including cocaine. While cocaine use is rarely associated with the Brugada sign without underlying BrS, associated ventricular tachyarrhythmias, cardiac arrest, a family history of SCD, and Southeast Asian ethnicity should prompt investigation of BrS. Additionally, it is necessary to gather further data to guide decisions in the context of cardiac transplantation in patients with BrS. 
